# Supplementary material for: Chromothripsis during telomere crisis is independent of NHEJ, and consistent with a replicative origin
Source: Genome Res. 2019 May;29(5):737–49. doi: 10.1101/gr.240705.118 (PMC6499312; doi:10.1101/gr.240705.118)
Supplement: Supplemental Material [file supp_gr.240705.118_Supplemental_file_1.zip › contigs/annotated_contigs/DB114/contig.2.DB114_length_242_mean_cov_9.81818181818.docx]

**DB114_length_242_mean_cov_9.81818181818**

```
TAGGGTTCAAGTGATTCTTCTGCCTCAGCCTCCCGAGTAGCTGAGACTACAGGCTCATGCCACCACACCCAGCTAATTTTTGTATTTTT
 >chr2:127394444-127394570 - E=1e-64
AGTATAGACAGGGTTTCACCATGTTGGCCAGGAT|AGT|TTCGCCTAAGGAAGAATATGGTTTTCTTTGCTTTTTGAACATTACATAAG
                                   >chr2:127394227-127394346 - E=2e-60
TGGAATCATATAGTGTCTACTTATTGGAGTCTGGCTTTTCTCACTCAACTTATGTCCATAAAATTA
```
